# Electrocatalytic Reaction Induced Colloidal Accumulation: The Role of Dielectrophoresis

**DOI:** 10.1021/acs.langmuir.1c01938

**Published:** 2022-03-01

**Authors:** Abimbola A. Ashaju, Jeffery A. Wood, Rob G. H. Lammertink

**Affiliations:** Soft Matter, Fluidics and Interfaces, MESA+ Institute for Nanotechnology, University of Twente, 7522NB Enschede, The Netherlands

## Abstract

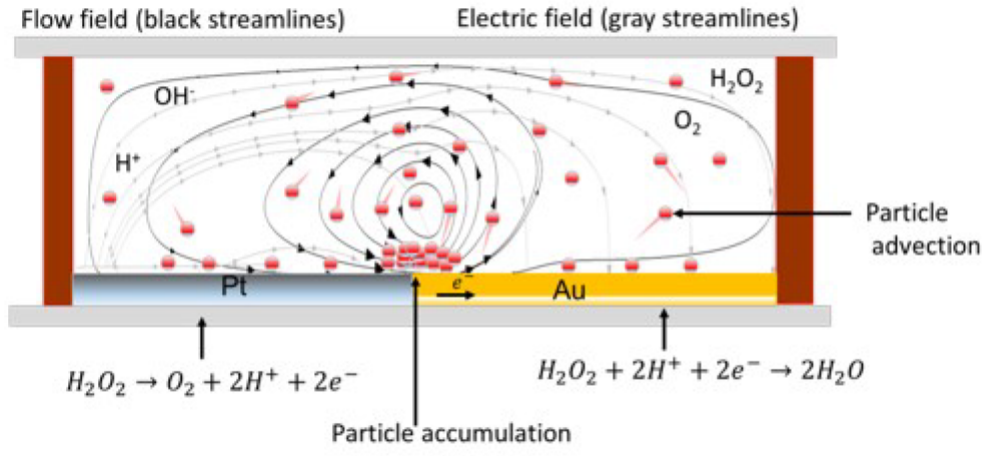

A surface-driven flow is generated during the electrocatalytic reaction of a platinum–gold bielectrode within hydrogen peroxide. This flow can be experimentally visualized and quantified using micrometer-sized particles that are transported by a flow field. Tracer particles, which possess an inherent surface charge, also interact with the induced electric field and exhibit a collective behavior at the surface of the electrodes where they accumulate. The underlying mechanism for the accumulation dynamics demonstrated by these catalytic pump systems has so far been lacking. In this work, the accumulation dynamics and kinetics were experimentally investigated. With use of numerical simulations, we demonstrate that the self-driven particle accumulation is controlled by a positive dielectrophoretic force, mediated by the reaction-induced electric and flow field. These results contribute to the fundamental knowledge on immobilized bimetallic systems.

## Introduction

**Figure 1:**
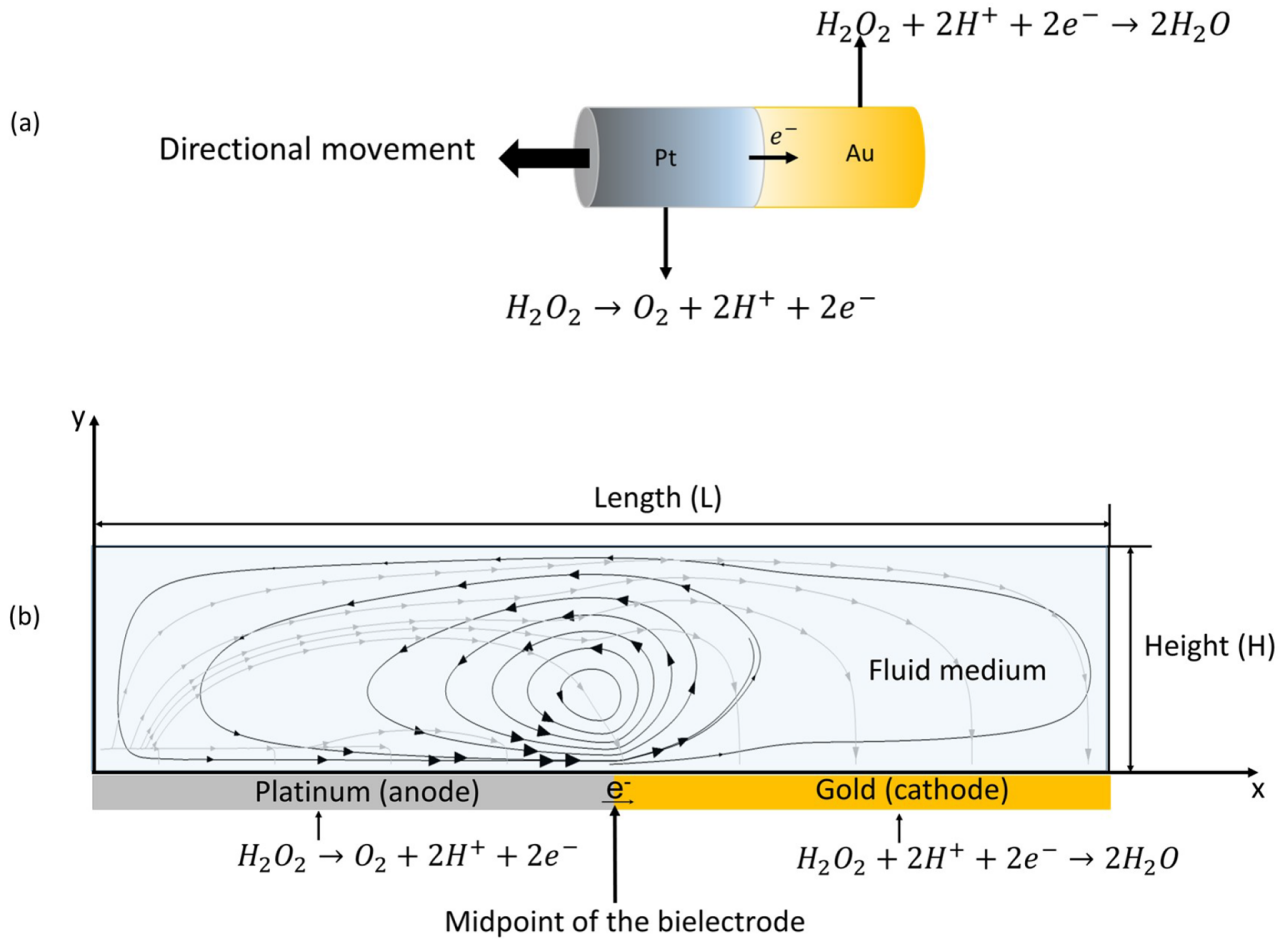
Schematic diagram of (a) a Pt–Au bimetallic nanorod and (b) a Pt–Au bimetallic micropump in hydrogen peroxide solution, showing the fluid flow (black streamlines) generated and the self-induced electric field (gray streamlines) during electrocatalytic decomposition of H_2_O_2_. The height of the channel is denoted by *H*, and *L* is the length of the bielectrode.

Several biological systems exist in nature that convert chemical energy to execute high-precision translational and rotational autonomous motion. This has inspired a plethora of research and developmental activities on artificial micromachines, aimed at understanding and replicating the complex functionalities presented by these biological motors.^1−4^ A good example is the autonomous locomotion of a catalytic nanorod that functions primarily on the conversion of chemical energy harvested from its surrounding fluid media (see Figure 1a). The Pt–Au bielectrode catalyzes the decomposition of hydrogen peroxide via surface reactions, leading to the establishment of concentration and electrical potential gradients that propel the nanorod.

Previously, the locomotion of catalytic bimetallic motors was attributed to mechanisms based on interfacial tension^5^ and oxygen bubble recoil.^6^ Later, a consensus was reached on self-electrophoresis as the main driving force, which is controlled by localized concentration and electrical gradients and fueled by the chemical conversion of the surrounding electrolyte. The phoretic movement of the bimetallic motor is powered by a self-induced electric field in conjunction with the proton gradient that is generated during the electrochemical reaction, hence the name self-electrophoresis.

Paxton et al.^7^ initially demonstrated self-electrophoresis using striped bimetallic motors, which catalyzes the decomposition of hydrogen peroxide through surface reactions and creates electric field **E** that propels the movements of the bimetallic motors (Figure 1a). The resulting electric field can induce particle migration whose velocity **u**_ep_ can be expressed by the Helmholtz–Smoluchowski equation **u**_ep_ = μ_e_**E**, where μ_e_ is the electrophoretic mobility of the particle. The self-electrophoretic mechanism requires a net charged layer surrounding the rod, which is generated by the electrochemical reactions that generate the asymmetric distribution of charged intermediates. The transport of the ionic species is governed by diffusion, migration, and convective fluxes. The charge density combines with a self-induced electric field to create a body force that powers the rod movement.^8^ These fields, which are coupled, can be solved numerically.^9^ Moran and Posner^10^ developed a comprehensive self-electrophoretic numerical framework for a bimetallic motor by solving the coupled Poisson–Nernst–Planck and Stokes equations, where the surface reaction and fluxes can be accounted for using the simplified Frumkin corrected Butler–Volmer equations. The velocity of the bimetallic motor showed dependence on several parameters including the concentration of the fuel and conductivity of the medium.^11^

Kline et al.,^5^ inspired by Paxton’s Au–Pt bimetallic motors,^7^ patterned a Au–Ag bielectrode on a substrate and contacted this with a hydrogen peroxide solution. This configuration immobilizes the bimetallic motor and restricts its motility during the electrochemical reaction with hydrogen peroxide. On the basis of the principle of Galilean relativity, a fluid flow was generated within the immediate surroundings of the bielectrode system. The immobilized bimetallic motor is also termed a micropump because of its fluid-pumping capability (see Figure 1b).

A catalytic micropump is a form of chemically powered pumps that exhibits diffusio-electroosmosis phenomena. Other types of chemically powered pumps can be activated through other mechanisms such as enzymatic reactions,^12,13^ photochemical reactions,^14^ and diffusioosmosis.^15^ A broad and in-depth review of chemically powered micropumps can be seen in Esplandiu et al.^16^ and Zhou et al.^17^

The fluid flow is driven by an electric body force generated by an induced electric field acting on a net charge distribution, which arise from a proton concentration gradient that is established during the electrochemical reaction involving the decomposition of hydrogen peroxide. The reaction proceeds via oxidation and reduction pathways where oxidation occurs at the anode (platinum), leading to the generation of protons and oxygen, and reduction occurs at the cathode (gold), involving the consumption of protons and production of water. Early catalytic micropump designs were fueled by the decomposition of hydrogen peroxide. However, its low-conversion efficiency limits its potential for practical applications,^18^ which have necessitated the search for alternative electrolytes. Hydrazine derivatives consisting of N_2_H_4_ and N_2_Me_2_H_2_ have been studied in conjunction with Au–Pd bielectrodes.^19^ More recently, halogen-based systems have been proven to be efficient and superior in performance compared to other forms of fuel-based systems.^20^ The chemomechanical actuation for these systems is similar to H_2_O_2_, where the decomposition of the fuel creates a gradient of charged intermediates that drive the fluid motion.

The electrochemical mechanism involving the decomposition of hydrogen peroxide has been established as the dominant mechanism through electric current measurement^21^ and mixed potential theory from Tafel plots.^22^ The proton gradient has been studied and confirmed using numerical modeling^10,23,24^ and fluorescence imaging.^25,26^

The induced fluid flow pattern is typically driven from the anode to the cathode and recirculated to the upper region within a confined space because of continuity. The fluid velocity **u** is impacted by the concentration and bulk pH of the electrolyte. The direction of the flow can be reversed from the cathode to the anode. This can be reaction-driven under lower anodic reactive regimes^23,27^ or by surface modification of the electrodes, which vary the zeta potential and impact the flow direction according to the Helmholtz–Smoluchowski formula ,^28^ where ζ_m_ is the electrode’s zeta potential.

The flow field is commonly visualized with tracer particles that passively follow the fluid flow. An example of commonly used tracer particles include polystyrene beads, which can be functionalized with amine or carboxylate to tune the zeta potential of the particle.^5,21^ Such particles have been used to quantify the fluid velocity magnitude. The observed particle velocity magnitude **u**_p_ consists of the fluid flow component **u** combined with the electrophoretic velocity of the particle **u**_ep_. The electrophoretic velocity is generated as a result of the interaction between the surface charge of the particles and the induced electric field. Decoupling these two velocity components will yield the actual magnitude of catalytically induced fluid flow. This can be achieved by using the two-particle correlation that involves the use of two tracers having different electrophoretic mobilities under the exact experimental conditions.^25,29^

In addition to particle transport by advection and electrophoresis, dynamic interactions and patterns have been observed for the tracer particles with electrocatalytic micropumps^5,19,25^ and micromotors.^18^ Wang and his team^18,30^ reported on the attachment of particles on bimetallic (Au–Pt) and trisegmented nanorods (Au–Ru–Au). The collective dynamics between these two classes of objects was attributed to an electrostatic force that is generated by the electric field.^31^ As soon as a moving nanorod approaches the charged particles, the particles are attracted toward the rod, leading to aggregation and formation of raft assemblies on the nanorod. Their modeling approach considered the nanorod as being uncharged, whereas the shear plane potential was made to float.

The dynamic interactions between an immobilized bimetallic system and colloidal particles occur irrespective of the combination of metals (Au–Pt, Au–Pd, Au–Ag, and Au–Cu) and fuel (H_2_O_2_, N_2_H_4_, and HCl) used in powering the system.^5,19,32−34^ In most cases, particles are observed to accumulate at the junction of the connected electrodes. The consensus for this behavior is that the particle attachment is primarily driven by an electrohydrodynamic force. The induced flow transports the particles toward the surface of the electrodes where they become trapped and accumulate under the influence of the self-generated electric field.

The directed transport of colloidal particles has been demonstrated for applications involving cargo transport^35^ and biological assays,^36^ biosensors,^37^ and optics.^38^ In the aforementioned applications, the collective transport and accumulation kinetics of the particles are controlled by an externally applied field. In the case of immobilized electrocatalytic systems where the particle collective dynamics is self-driven, it is crucial to fully understand the underlying mechanism to fully exploit this behavior for relevant applications.

This article investigates the role of the dielectrophoretic (DEP) force toward the accumulation of charged particles in a bimetallic catalytic system. We present quantitative and numerical analyses that pertain to the agglomeration dynamics of particles at bimetallic electrocatalytic junctions for different time scales and electrolyte concentrations. The results show that the dielectrophoretic force, mediated by the electric field in conjunction with the surface-induced flow, plays a dominant role in particle agglomeration, which is observed for immobilized electrocatalytic systems.

## Theory and Numerical Modeling

Dielectrophoresis is an electrokinetic phenomenon exploited for several lab-on-chip applications that range from sorting, separation, manipulation, and concentration of microparticles, in addition to cells and viruses.^39−42^ When a dielectric particle is exposed to a nonuniform electric field, it becomes polarized, and as a result, the surface charges are reoriented, inducing a dipole.^43^ The interaction between a dipole and an electric field gradient creates a dielectrophoretic force, **F**_DEP_.^44^ For the immobilized electrocatalytic system, the dielectrophoretic force is strongly dependent on the reaction-induced electric field, which can be directly influenced by the geometry of the electrodes.

**Figure 2:**
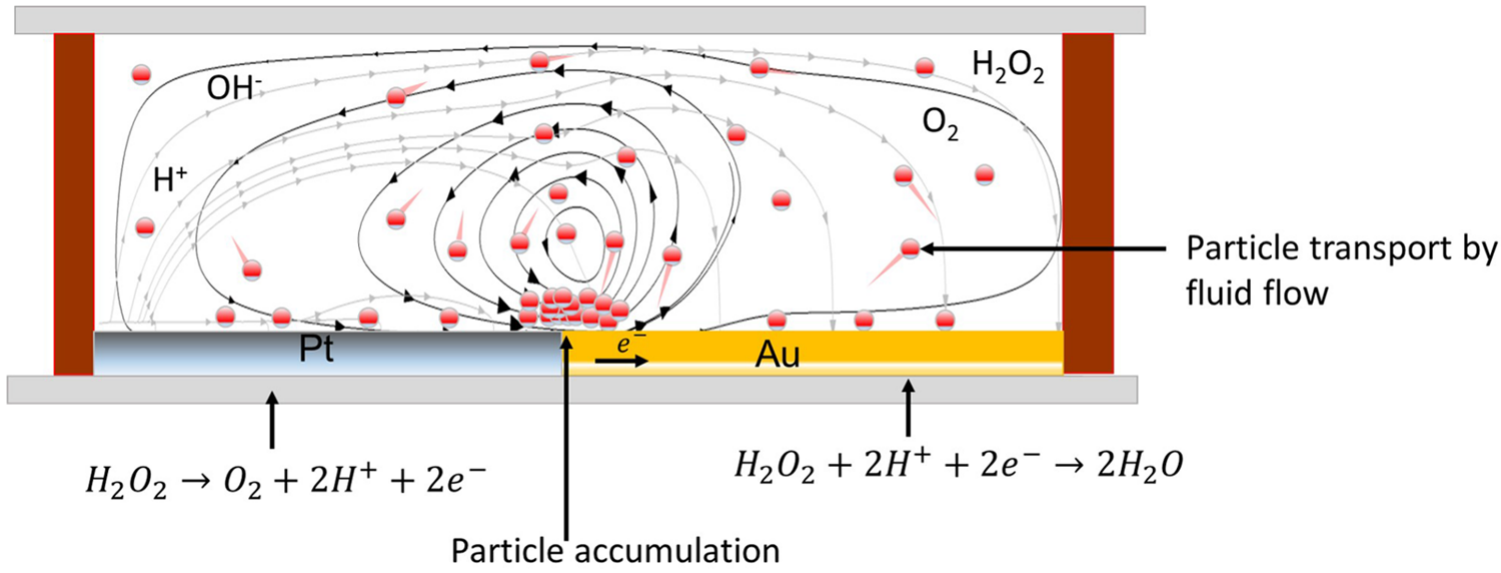
Schematic diagram of the Pt–Au microreactor, which drives the movement and accumulation of colloidal particles during electrocatalytic reaction. The black streamlines represent the fluid flow, and the gray streamlines refer to the electric field. The colloidal particles are advected by the fluid flow driven by the electric field. The electric field causes the dielectrophoretic force that influences particle accumulation at the junction of the bielectrode.

To understand and predict the interaction between the dielectrophoretic force and the tracer particles, we developed 2D models including Pt–Au bielectrodes. The bimetallic and interdigitated electrodes are immersed into a solution of hydrogen peroxide and tracer particles and confined within a chamber of depth *h*. Figure 2 presents a schematic diagram of an immobilized Pt–Au bielectrode that catalyzes the decomposition of hydrogen peroxide via redox reactions. Oxidation occurs at the Pt end, which generates protons, oxygen molecules, and electrons. The electrons are directly transferred to Au to complete the circuitry. During the reduction reaction at the Au electrode, protons are consumed and combined with hydrogen peroxide to create water. The flux of protons between the electrodes establishes a proton concentration gradient that generates an electric field **E** and combines with the net charge distribution to form a body force that drives the interfacial fluid flow. The flow, which was visualized with tracer particles, is driven from platinum toward gold under normal electrocatalytic steady-state conditions and recirculates within a closed system.

The charged particles that are close to the surface of the bielectrode are trapped and concentrated at the junction of the bielectrode. The dielectrophoretic force acting on the particle is expressed as^40^
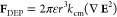
1where ε is the fluid’s permittivity, *r* is the radius of a particle, and *k*_cm_ is the Clausius–Mossotti (CM) factor that is defined in terms of the complex permittivity of the particle ε_p_* and the fluid medium ε_f_* respectively as
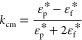
2Under the influence of a direct current (DC) field or at frequencies below 100 kHz, the CM factor can be approximated in terms of the electrical conductivity of the fluid medium σ_f_ and the particle σ_p_,^45,46^ given as
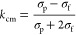
3If σ_p_ > σ_f_, the dielectric particle will be attracted toward the region of high electric field strength (positive dielectrophoresis), and if σ_p_ < σ_f_, the particles are drawn to a weak electric field region (negative dielectrophoresis). According to O’Konski,^47^ the conductivity of a particle is defined as
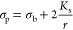
4where *K*_s_ denotes the surface conductance, which is about 1 nS for latex particles,^48^ and σ_b_ represents the bulk conductivity of polystyrene, which has been proven to be σ_b_ ≈ 0.^47,49^ The surface conductance with particle radius *r* = 0.5 μm results in an effective particle conductivity of 40 μS/cm. The conductivity of the fluid medium was measured in the range of 5–30 μS/cm, leading to positive dielectrophoresis.

### Governing Equations

Here, we present the governing equations that resolve the ionic species concentration field, the induced electric field, the resulting flow field, and the trajectories of the colloidal particles. Moran and Posner^10^ used a similar approach to model the locomotion of catalytic nanomotors.

Under the assumption of a dilute solution limit, the concentration of ionic species (H^+^, OH^–^, Cl^–^) is solved by the Nernst–Planck equation,
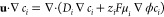
5where **u** represents the fluid velocity, *c*_*i*_ is the molar concentration for the *i*th species, having a diffusion constant *D*_*i*_ with valence *z*_*i*_, and ϕ is the electrostatic potential. The potential distribution in conjunction with the space charge density ρ_e_ is described by the Poisson equation,
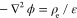
6where . The gradient of the potential distribution gives rise to the electric field described as **E** = −∇ϕ.

The Stokes and continuity equations describe the fluid flow for an incompressible Newtonian fluid operating under a lower Reynolds regime (*Re* ≪ 1),
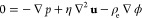
7
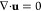
8The movement of the particles is described by Newton’s law of motion,
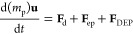
9where *m*_p_ is the mass of the particle and **F**_d_ is the drag force that acts on the particles of radius *r* moving with velocity **u** through a peroxide solution with viscosity η,^50^ given as
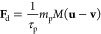
10τ_p_ is the response time of the particle velocity in seconds and **v** is the velocity of the particles (m/s). *M* is a correction for the wall effects given as
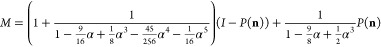
11where **I** denotes the identity matrix, **P**(**n**) defines the projection operator onto the wall normal **n**, , and *l*_w_ is the distance from the center of the particle to the nearest wall.^51^

The electrophoretic force **F**_ep_ is defined as
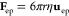
12where  is the electrophoretic velocity of the particles.^52^ The zeta potential of the particles is denoted by , which was measured to be −45 mV.^27^

The drag, electrophoretic, and dielectrophoretic forces combine to a net resultant force on the particles, resulting in its acceleration. The particles in this case are assumed to be spherical in shape. The gravitational force is neglected because the particles are assumed to be neutrally buoyant.

### Boundary Conditions

A no-slip condition, **u** = 0, is applied to the top and bottom boundaries. A symmetry boundary condition is applied on the left and right walls of the bimetallic system model. For the interdigitated electrode, we imposed a periodic boundary condition on the left and right walls to indicate a repeated pattern for Pt and Au for which we simulate a single period. For the potential boundary conditions, we solved explicitly the diffuse part of the electric double layer. The potential drop across the Stern layer is described by
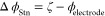
13which scales with the ratio of the Stern layer length *L*_Stn_ to the Debye length *L*_D_ as well as the zeta potential of the electrode ζ_electrode_ as
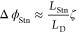
14where  is usually hundreds of nanometers,  is the thermal voltage, *T* is the temperature, *F* is the Faraday constant (*F* = 96485 C mol^–1^), *R* is the universal gas constant (8.314 J/K·mol), and *c*_*∞*_ represents the bulk proton concentration. For an electrolyte having a low salt concentration, , and as a result Δϕ_Stn_ in eq 13 becomes negligible. Equation 13 is applied at the surface of Pt and Au (*y* = 0) in terms of their respective zeta potentials as ϕ_Pt_ = ζ_Pt_ and ϕ_Au_ = ζ_Au_. The zeta potentials for platinum and gold electrodes on a glass substrate has been determined experimentally for bulk pH 3–10 (see ref (27)) and was coupled to eq 13 using an interpolation function. The upper boundary of the model is set as .

The reactive current that runs through the electrodes has been experimentally measured^27^ and is described by the Frumkin–Butler–Volmer equation^53,54^
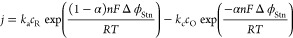
15from which the reaction kinetics and proton flux are determined. The proton reaction flux is expressed from the measured current density, *j* = *i*/*nFAz*, where *A* is the surface area of the bielectrode, *n* is the number of electrons transferred during the reaction, *c*_O_ and *c*_R_ are the generalized oxidation and reduction reactants concentrations, and *k*_a_ and *k*_c_ are the anodic and cathodic rate constants, respectively. Following the approach employed by Moran and Posner^10^ for the catalytic nanomotors, we considered the irreversible electrochemical reactions for both electrodes to proceed in the forward direction under the Tafel regime. Hence, the proton reaction flux for the anodic reaction is expressed as
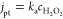
16and the cathodic reaction is given as
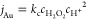
17The fluxes of anions (OH^–^, Cl^–^) at the surface of the electrodes (*y* = 0) are set to zero since they do not participate in the electrochemical reaction.

The particle tracing module in COMSOL Multiphysics V.5.5, a finite element based commercial solver, was used in resolving the particles trajectories.^55^ To simulate the trapping and accumulation of particles at the electrode’s surface, a stick boundary condition **u** = 0 was imposed on the electrodes, which results in the stoppage of the particle’s trajectories as soon as they contact the electrode. The number of accumulated particles at the surface of the electrodes was determined by imposing particle counter boundary conditions in the particle tracing module, in the form of a logical expression that is effective when the particles contact the electrodes and computes the total number of accumulated particles at every time step.

### Numerical Method and Simulation

The 2D models were partitioned into subdomains to implement a user-controlled nonuniform mesh that consists of triangular elements. A uniform mesh was imposed at the tangential *x*-direction within the lower part of the model, using mapped meshing control. This results in refinements that resolve the electric double layer and other gradients at the surface of the electrodes.

The governing equations are solved using COMSOL Multiphysics V.5.5. At the beginning of the simulation, the steady Nernst–Planck and Poisson equations are computed without the flow field to generate the initial conditions for the system, which were used in solving the coupled governing equations sequentially until the solution converges. The results are implemented as initial values for a time-dependent solver used in resolving the particle trajectories, where time stepping is performed using a generalized alpha implicit method.

## Experimental Method

**Figure 3:**
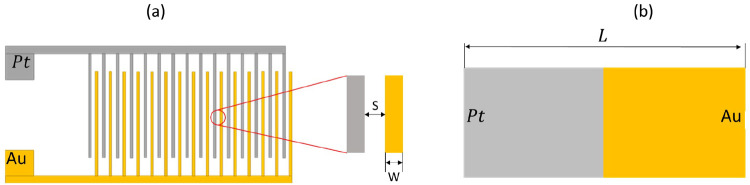
Schematic diagram of the Pt–Au microreactors. (a) Pt–Au interdigitated electrode with interspacing *S* = 50 μm, electrode width *W* = 100 μm, and total surface area *A* = 9.08 × 10^–6^ m^2^. (b) Pt–Au bimetallic electrode of length *L* = 10 mm.

The colloidal accumulation experiments were performed using platinum–gold bielectrodes that are immobilized on glass substrates. The bielectrodes were fabricated as follows: a positive resist was spin-coated on a glass wafer and then was exposed to UV light through a photomask and development. Both platinum and gold electrodes were sputtered on tantalum (5 nm), which was predeposited on the glass wafer (MESA+ nanolab cleanroom in-house equipment, “TCOathy”). Undesired metal residues were removed during the liftoff process by ultrasonification in acetone. Both Pt–Au interdigitated (IDE) electrodes and Pt–Au bimetallic electrodes were fabricated by this method (see Figure 3).

The interdigitated electrodes were bonded to a printed circuit board, thereby forming a configuration that allows us to control the electrochemical reaction. For the bimetallic electrodes, Pt and Au constitute a galvanic pair that maintains continuous contact with each other. Hydrogen peroxide (Sigma-Aldrich) solutions of known concentrations were prepared in Milli-Q water (ρ > 18 MΩ·cm). The pH of the resulting solutions were adjusted accordingly using HCl in the range pH 5–6, and the conductivity was measured with a conductivity probe (WTW Cond 3110, Weilheim, Germany) to be in the range of 5–30 μS/cm. Particle dispersions were prepared by seeding the hydrogen peroxide (10 mL) solution with 2.5 wt % (1 μL) fluorescent microparticles (PS-FluoRed-Fi329 by Microparticles GmbH), 1 μm in diameter, and introduced into a confined hybrid chamber that contains the Pt–Au bimetallic electrode. The motion and accumulation of the particles are observed with an inverted optical microscope (Carl Zeiss Axio Observer Z1, 20×). Image frames were recorded with a CCD camera with a frame rate of 10 fps.

Image analysis was done using the open access software, ImageJ.^56^ The image frames were digitized by converting to 8 bits. The contrast and brightness were adjusted, and segmentation was performed to isolate the particles by subtracting the image background. The images were transformed to a binary form by applying a standard maximum entropy threshold. Particle analysis was performed using the “analyze” command, which determines the number of particles as well as the average size of the accumulated particles in each image frame.

## Results and Discussion

### Experimental Results

**Figure 4:**
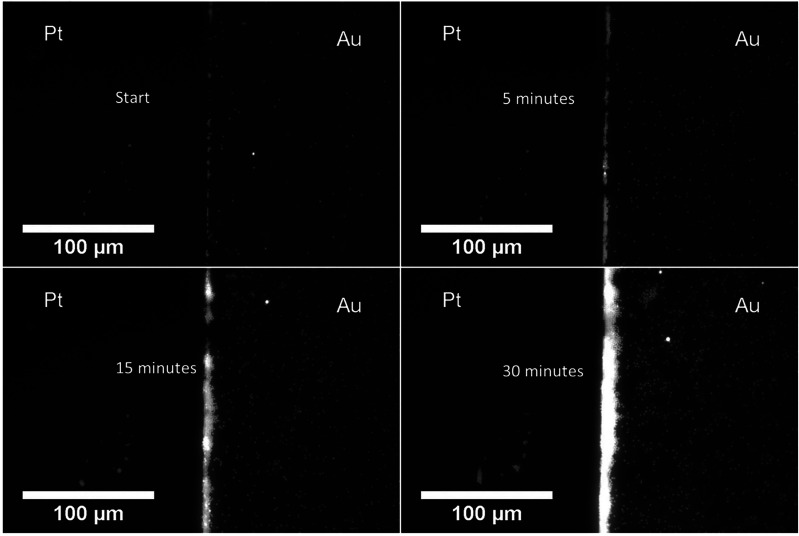
Time series colloidal accumulation at the junction of a Pt–Au bimetallic electrode.

The movement and collective dynamics of the particles are observed with optical microscopy. The tracer particles are transported near the surface of the electrodes (at a relative height around *y* = 10 μm) by the induced fluid flow from the anode (platinum) to the cathode (gold) (see Movie 1 Supporting Information). The particles close to the junction of the connected electrodes become trapped, and the population grows in clusters (see Movie 2 Supporting Information). Figure 4 shows the time series for the particle accumulation at the junction of a Pt–Au bimetallic electrode, which intensifies with time and slightly extends above the surface of the bielectrode.

**Figure 5:**
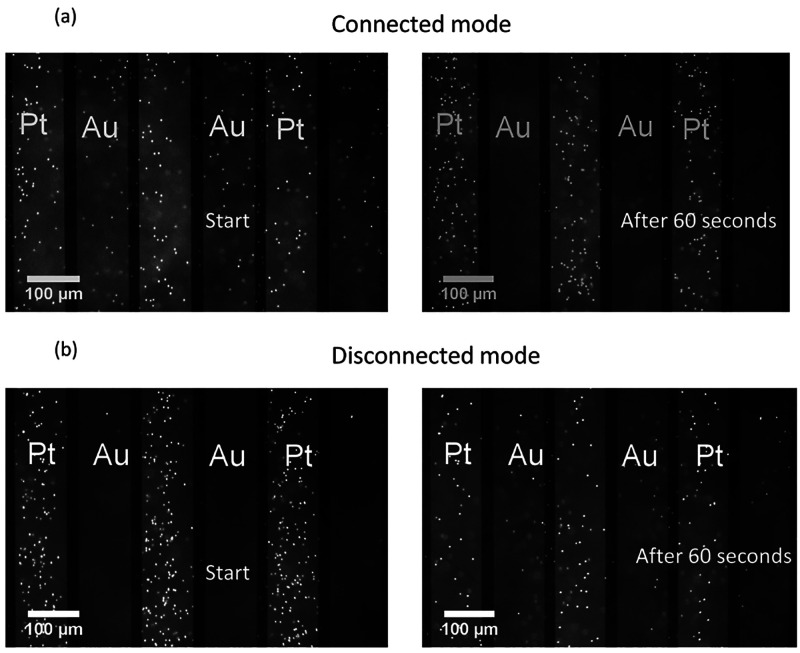
(a) Colloidal trapping at the platinum electrode during the connection mode. (b) Release of the particles during the disconnected mode.

The particle accumulation behavior was related to the reaction mechanism involving the catalytic decomposition of hydrogen peroxide by repeating the experiment using interdigitated electrodes, where the reaction is monitored and controlled by an external current measurement. During the connected mode, the particles close to the surface of the electrodes are migrating toward the platinum electrode where they maintain their Brownian motion (see Figure 5a, Movie 3 Supporting Information). At longer time scales, the particles are observed to accumulate and grow into clusters (see Movie 4, Supporting Information). As soon as the electrodes are disconnected, the particles are released from their trapped state and slowly disperse again into the bulk region (Figure 5b, Movie 5 Supporting Information). To understand what is happening, we refer to the electrochemical behavior of the Pt–Au interdigited electrodes in hydrogen peroxide solution.^27^ During the connected mode, a potential distribution is induced following the balanced reactive fluxes on the electrodes. The thus-generated electric field plays two main roles. First, it drives the fluid flow in conjunction with the space charge density in an electroosmotic fashion. Second, it interacts with the particles to create (dielectro)phoretic forces that affect the movement of the particles (Figure 2).

**Figure 6:**
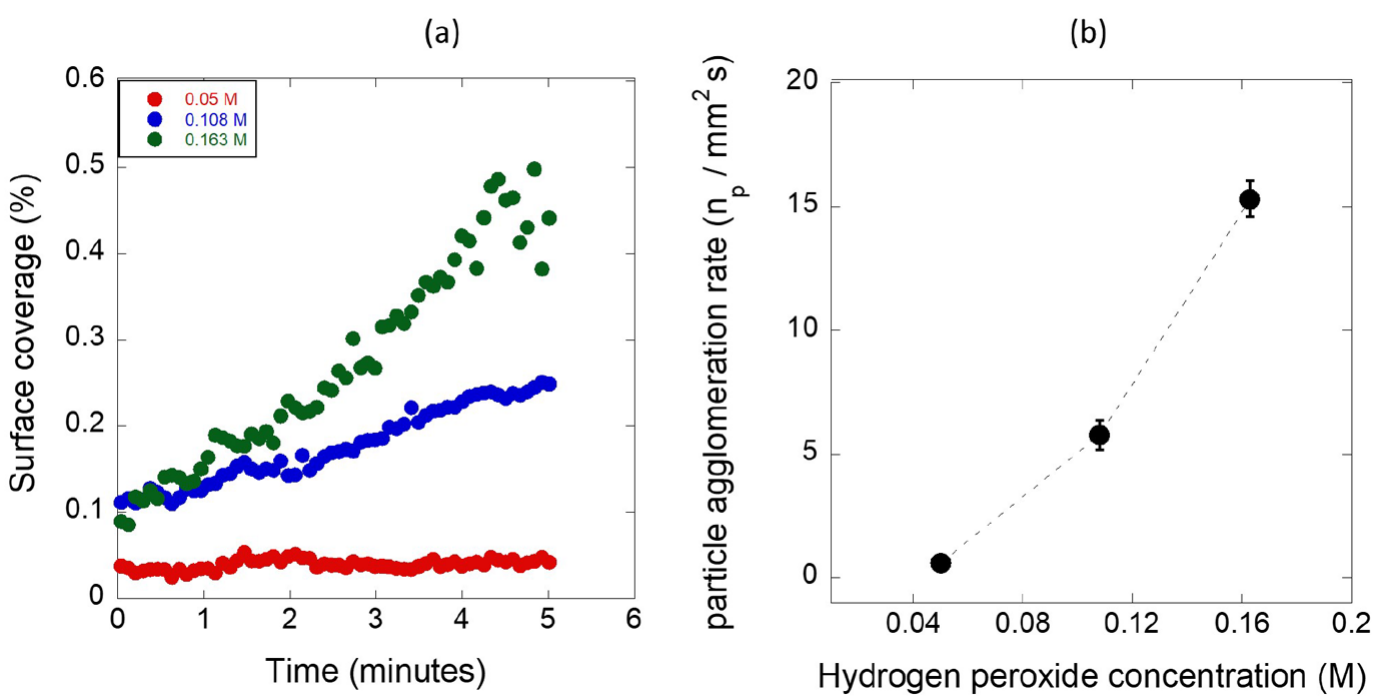
Effects of H_2_O_2_ concentration on (a) percentage surface coverage by the particles and (b) accumulation rate constant. Data points for the rate constant are averaged from three experiments. Dashed line serves as a guide to the eyes.

Now that we know that the particle accumulation dynamics is controlled by the reaction-induced electric field, we quantify the accumulation dynamics by estimating the surface coverage (the area occupied by the trapped particles vs the total surface area of the electrode) of the trapped particle monolayer formed during the first minutes of the accumulation process involving the Pt–Au bimetallic electrode. The quantification method is described in section S1 of the Supporting Information. The surface coverage is plotted as a function of time for different H_2_O_2_ concentrations (0.05, 0.108, and 0.163 M) and shown in Figure 6a. The clear trend depicts the increase in the accumulation rate with an increase in the H_2_O_2_ concentration.

To elucidate the influence of H_2_O_2_ on the accumulation kinetics, we determined the agglomeration rate constant from the number of trapped particles *n*_p_ versus time, which is fitted with a linear regression. This was evaluated during the first 5 min of the accumulation process before the particles form multilayers. Figure 6b suggests that the accumulation rate increased rapidly at higher peroxide concentrations owing to the increase in field strength due to the impact of the peroxide concentration on the electrochemical reaction rate.

### Particle Accumulation by DC Dielectrophoresis

A series of control tests was performed to ascertain the dominance of the dielectrophoretic force on the particles. This was done by varying the conductivity of the fluid that results in the variation of the CM factor and plotted against the particle agglomeration rate (see Figure S2.1, Supporting Information). As the conductivity of the suspending medium increases, the CM factor decreases and the particle accumulation rate driven by the positive DEP force reduces. This trend is also observed in the simulation results obtained by varying the conductivity of the fluid, where an increase in the conductivity of the fluid medium results in the reduction of trapped particles by the DEP force.

**Figure 7:**
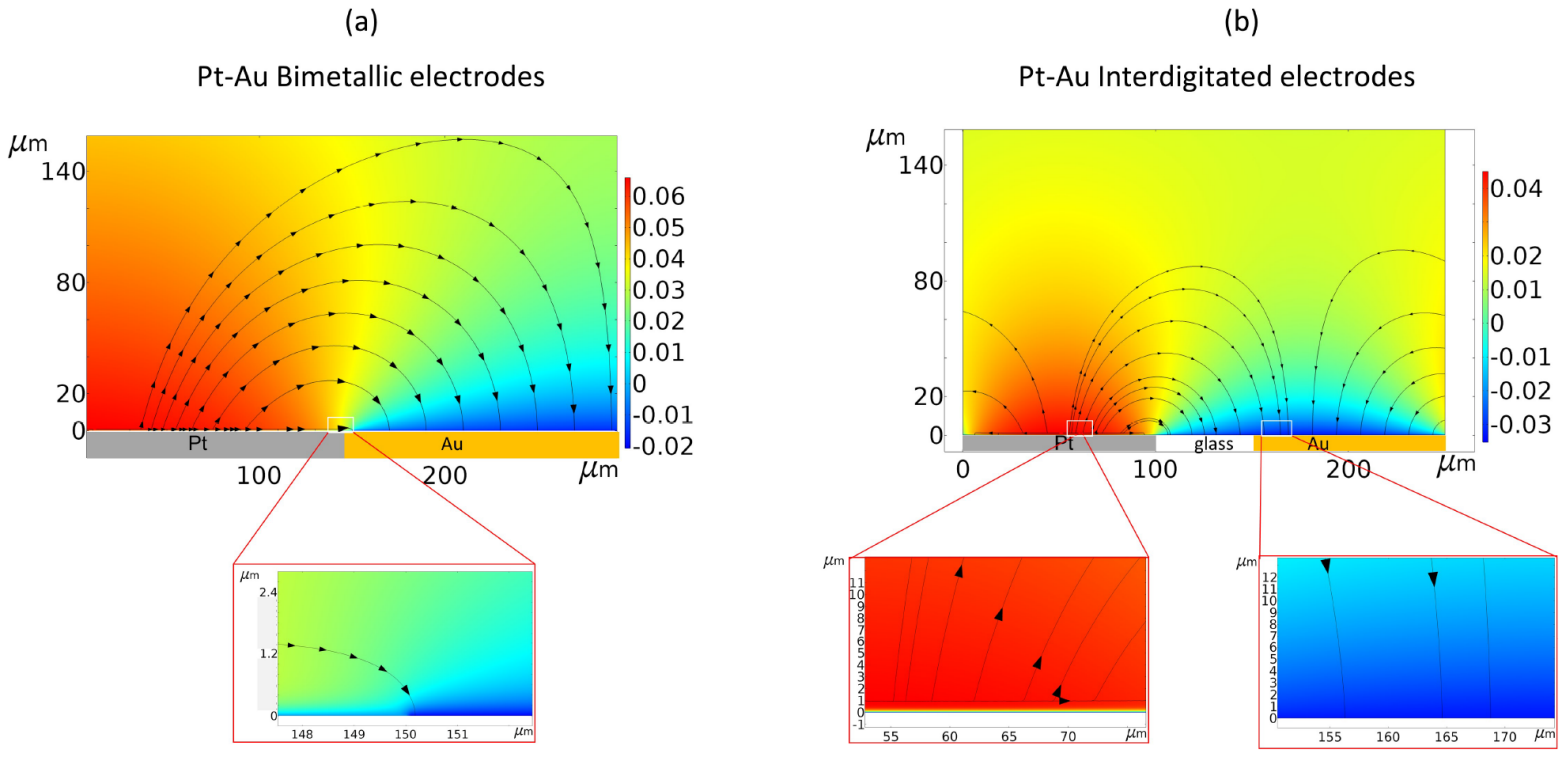
Electrocatalytically induced potential distribution (in volts and colorscale), overlaid with the electric field streamlines for (a) Pt–Au bimetallic electrodes and (b) Pt–Au interdigitated electrodes. Insets show a zoom on the potential field close to the surface of the electrodes. The simulation results are obtained for 0.163 M H_2_O_2_, pH 6.1.

Our numerical simulations provide more insights on the particle accumulation behavior at the surface of the electrodes. We start by analyzing the reaction-induced electric field that drives the particle accumulation. Figure 7 shows the electric field lines across the Pt–Au bimetallic and interdigitated electrodes, combined with the reaction-induced potential distribution (color) that was evaluated for 0.163 M H_2_O_2_. In both cases, the electric field is generated by the proton gradient that originates from the electrochemical reaction involving the decomposition of H_2_O_2_^26^ and extends from the double-layer region just after the surface potentials of the electrodes (see the insets of Figures 7a,b) to the bulk region. The reaction-induced potential acts to balance the reactive current from the electrodes through the variation of the proton concentration at the electrode’s surface.^23^

**Figure 8:**
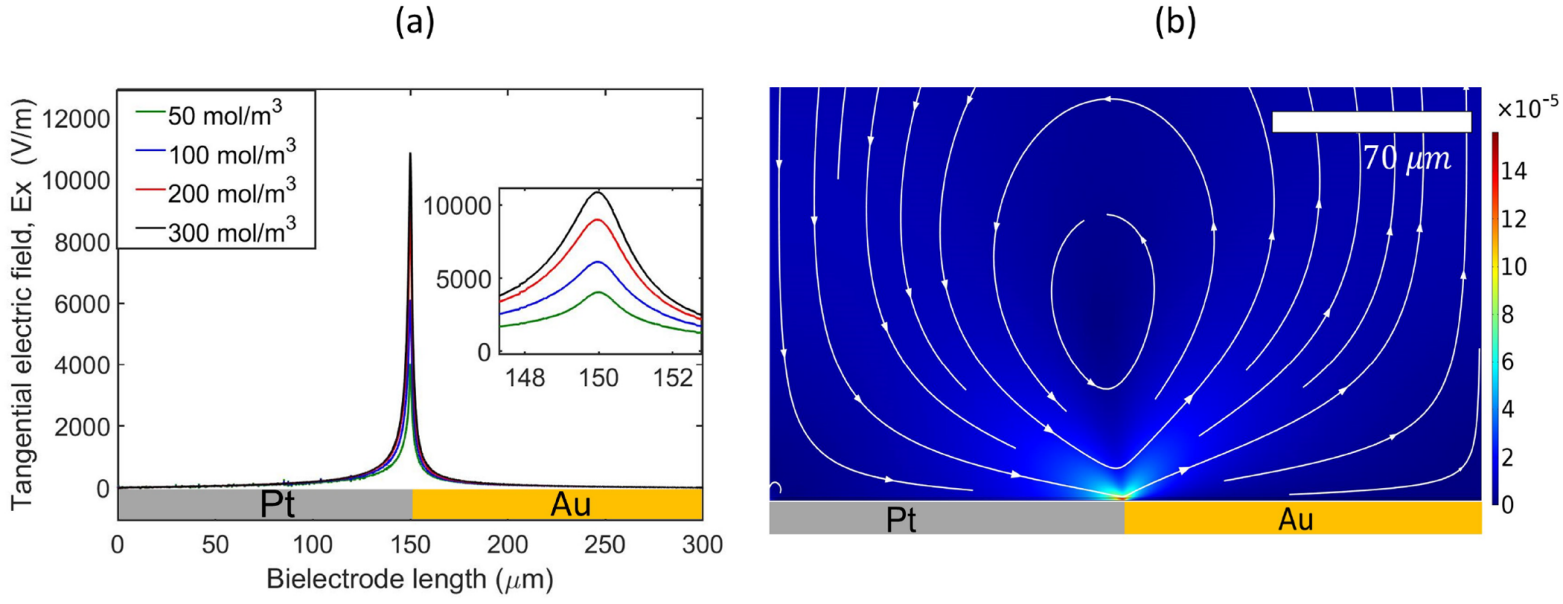
(a) Tangential electric field, **E**_x_, along the Pt–Au bimetallic electrode surface; (b) fluid flow pattern driven by the electric field gradient near the platinum–gold junction. The velocity magnitude is given in m/s and solved for 0.163 M H_2_O_2_, pH 6.1.

We see in Figure 7 that the electric field originates from platinum and the field line extends toward gold. The apparent source of the electric field, which is rooted at platinum, is the region where the field contributions from the reaction-induced proton gradient and surface charges intersect.^10^ The direction of the field lines is dictated by the proton current across the bielectrode. Figure 8a shows the tangential electric field profile along the length of the bimetallic electrode whose gradient is maximum at the junction where both electrodes maintain a continuous contact. The physical inhomogeneity at the midpoint increases the electric field strength that creates the DEP force. The electric field combined with the charge density drives the fluid flow that transports the particles near the surface of the electrode during recirculation (Figure 8b), within the range of the positive dielectrophoretic force where they become trapped and accumulate. The charged particles are capable of generating electroosmotic flow due to interactions between the induced electric field and the double layer of the surface of the particles,^57,58^ which act to entrain nearby particles on the electrode. But they were not computed in the model as they are hardly visible in this case (see Movie 1 and Movie 2 in the Supporting Information).

Different strategies have been previously adopted to increase the local field to concentrate particles within the high field region. A common method is to use insulating structures that generate spatial nonuniformity in the local electric field known as insulator-based dielectrophoresis (iDEP), which increases the intensity of the field that strongly affects the particles.^40,59,60^ In another instance, a scratch was introduced on the surface of an ITO electrode, which increased the local current density by a factor of 2 compared to that of unscratched electrodes, and promotes a higher field strength that traps colloidal particles within the scratched region.^61^

In the case of the interdigitated electrode, the tangential electric field is maximum at the end of the platinum electrode just before the spacing between Pt and Au, and the resulting flow streamlines that are driven by the field gradient build up from this region, which transport particles close to the platinum end of the interdigitated electrode where they eventually accumulate (see Figure S.3.1).

**Figure 9:**
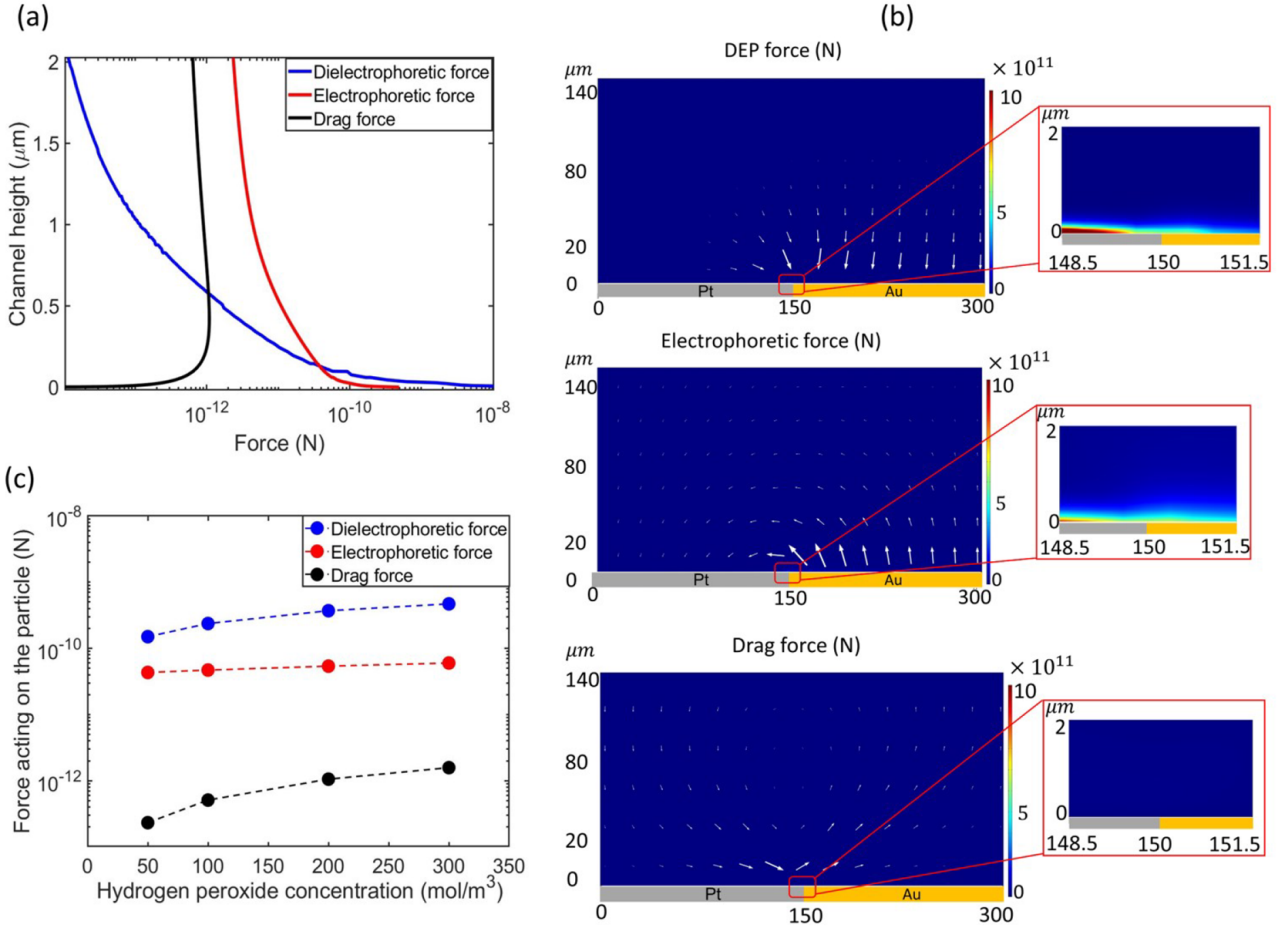
(a) Variation of the magnitude of the forces acting on the particles within a hydrogen peroxide solution, 0.163 M, along the *y*-axis from the midpoint of the bielectrode. (b) 2D vector field and arrow plot indicating the direction and magnitude of the respective forces. The insets highlight the respective force field near the surface region (c) The maximum of the forces acting on the particles versus the concentration of hydrogen peroxide evaluated along the *y*-axis from the midpoint of the bielectrode.

Figure 9a shows the average distribution of forces acting on the particles that are plotted from the bielectrode junction along the channel height. At several micrometers from the surface region, (*y* > 1 μm), the electrophoretic and drag forces dominate the DEP force. At *y* ≤ 1 μm, the DEP force is amplified by the high electric field strength and gradient and becomes more significant. At the surface region, the DEP force surpasses the other two forces by almost 3 orders of magnitude. As a result, the particles that are transported by the flow field are impacted by both the drag force and the electrophoretic force, and as soon as they are within the range of the positive DEP force, they are pulled toward the region of the maximum local electric gradient where they accumulate. The direction of these forces are highlighted by the vector plot in Figure 9b, and the magnitude of the DEP force is greater compared to that of the other two near the surface region of the bielectrode. The maximum of each force contribution was determined for different electrolyte conditions and plotted versus the concentration of H_2_O_2_ (Figure 9c). All forces were greater than the Brownian force acting on the particle, which was determined to be on the order of magnitude 10^–15^ N (see Supporting Information S4). We see that the positive DEP force exerts the most dominant force on the particles, which occurs at the bielectrode junction.

**Figure 10:**
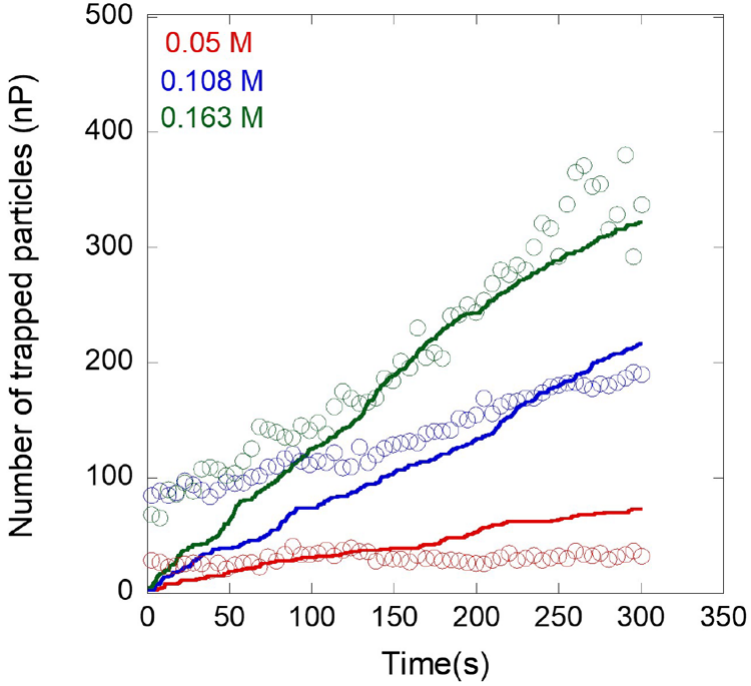
Comparison of the experimentally determined number of particles (open symbols) with simulation results (solid lines) for different H_2_O_2_ concentrations.

The accumulation statistics for the trapped particles at the junction of the Pt–Au bimetallic electrodes is determined numerically and compared to our experimental results for different H_2_O_2_ concentrations (see Figure 10). The accumulation proceeds at a vary fast rate, making it challenging to determine the trapped particle statistics for the first few seconds experimentally, which results in deviations at *t* < 100. In the numerical results the number of trapped particles and the agglomeration rate is influenced by the number of initial particles, *N*, used for the simulation (see Figure S2.2, Supporting Information S2); nonetheless, the numerical result matches well with the experimental data in Figure 10 when the initial number of particles is set to 1000. At longer time scales, the model is capable of highlighting localized particle trapping and accumulation exhibited by bimetallic catalytic micropumps.

## Conclusion

In this article, we studied the dynamics of the reaction-driven colloidal trapping and accumulation by a Pt–Au bimetallic catalytic system. We showed that the accumulation of particles occurs under the influence of a positive dielectrophoretic force that is mediated by a self-induced electric field. The particle accumulation kinetics were studied experimentally and found to scale with the concentration of the fuel that drives the electrocatalytic reaction, which generates the induced electric field. Numerical modeling elucidates the role of the drag and electrophoretic force that directly influences the movement of the particles further away from the surface region, while they accumulate under the effect of a positive dielectrophoretic force at the surface. Our combined experimental and numerical approaches shed light on the underlying mechanism of the reaction-induced particle accumulation and advance the knowledge of the transport mechanism by immobilized electrocatalytic systems.

## Supporting Information Available

Particle agglomeration quantification method; DC dielectrophoresis control tests and results; simulation results showing field quantities (velocity, potential, and dielectrophoretic force) for the interdigitated electrodes; Brownian force acting on particles (PDF)
Movie 1: Transport of colloidal particles by the catalytically induced flow field from platinum to gold (MP4)
Movie 2: Time lapse for particle trapping and accumulation at the junction of the Pt–Au bimetallic electrode (MP4)
Movie 3: Accumulation of particles on the surface of the platinum electrode during the connected mode for the interdigitated electrodes (MP4)
Movie 4: Accumulation of particles into clusters at longer time periods (MP4)
Movie 5: Behavior of the particles under the disconnected mode of operation; the trapped particles break into singlets and slowly disperse back into the bulk region (MP4)
