# Supplementary material for: Electrocatalytic Reaction Induced Colloidal Accumulation: The Role of Dielectrophoresis
Source: Langmuir. 2022 Mar 1;38(10):3040–50. doi: 10.1021/acs.langmuir.1c01938 (PMC8928468; doi:10.1021/acs.langmuir.1c01938)
Supplement: Supplementary file 1 — la1c01938_si_001.pdf [file la1c01938_si_001.pdf]

Electrocatalytic reaction induced colloidal accumulation: The role of dielectrophoresis

Abimbola A.Ashaju, Jeffery A. Wood, and Rob G.H. Lammertink*

Soft Matter, Fluidics and Interfaces, MESA+ Institute for Nanotechnology, University of Twente, 7522NB Enschede, The Netherlands

E-mail: r.g.h.lammertink@utwente.nl

S1 Quantification of particle accumulation

The percentage surface coverage is defined as

$$\frac{A_p}{A_s} \times 100 \tag{S1.1}$$

where $A_s = L \times W$ is the surface area of the bielectrode (figure S1.1), L is the length of the bielectrode ($L = 300\mu m$), and W is the width ($W = 200\mu m$). A_p denotes the total area occupied by the trapped particles, $A = \Sigma \pi r^2$, where r is the radius of a particle. The number of trapped particles (n_p) is defined as

$$n_p = \frac{A_p}{\pi r^2}. \tag{S1.2}$$

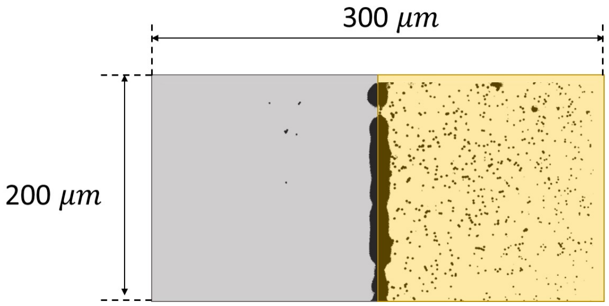

Figure S1.1: Schematic that describes the surface area of the bimetallic electrode

S2 DC dielectrophoresis control tests

Control tests were performed experimentally and numerically to confirm the DEP force as the dominant mechanism that drives the particle accumulation dynamics. Figure S2.1 highlights the impact of the DEP force on the particle accumulation rate influenced by the variation of the CM factor. The CM factor is one of the key parameters that contributes to the magnitude of the DEP force.

The initial number of particles, N, used in the simulation was varied to determine its effect on the particle trapping mechanism. As shown in Figure S2.2, the particle trapping intensifies with an increase in the initial number of particles.

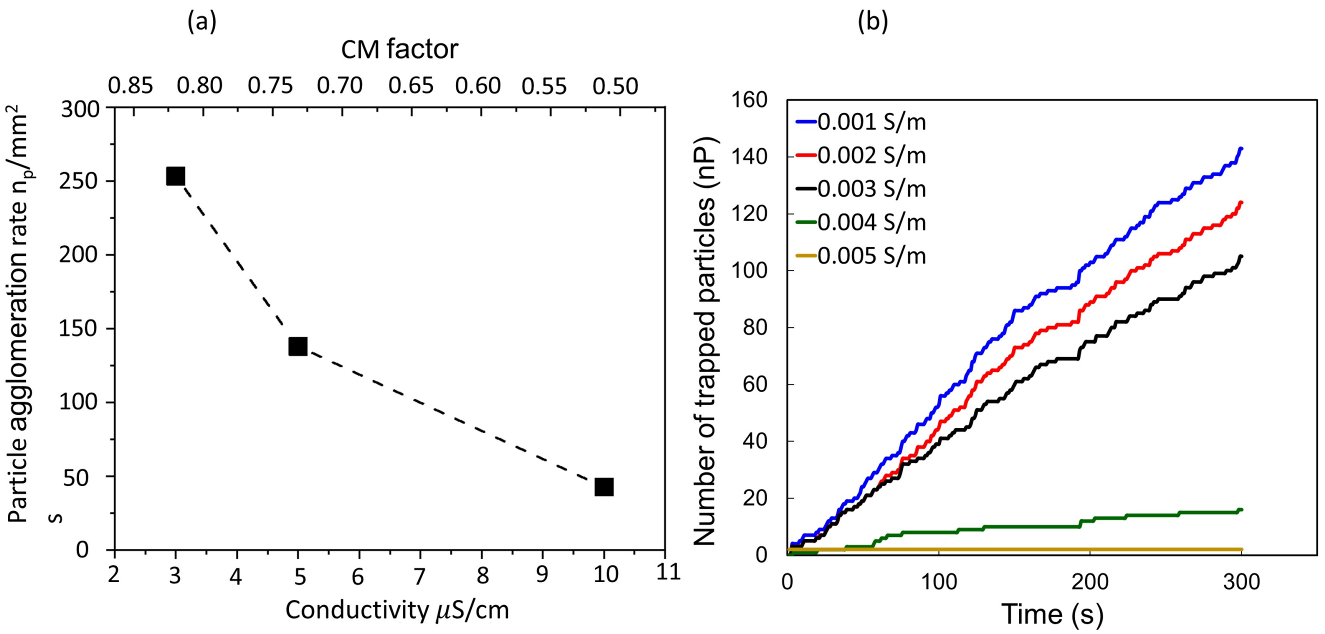

Figure S2.1: Experimental and numerical results that highlights the dominance of DEP force towards particle accumulation (a) Experimental results showing the variation of the CM factor and the conductivity of the fluid medium with the agglomeration kinetics of the particles (b) Effects of the fluid conductivity on the number of trapped particles.

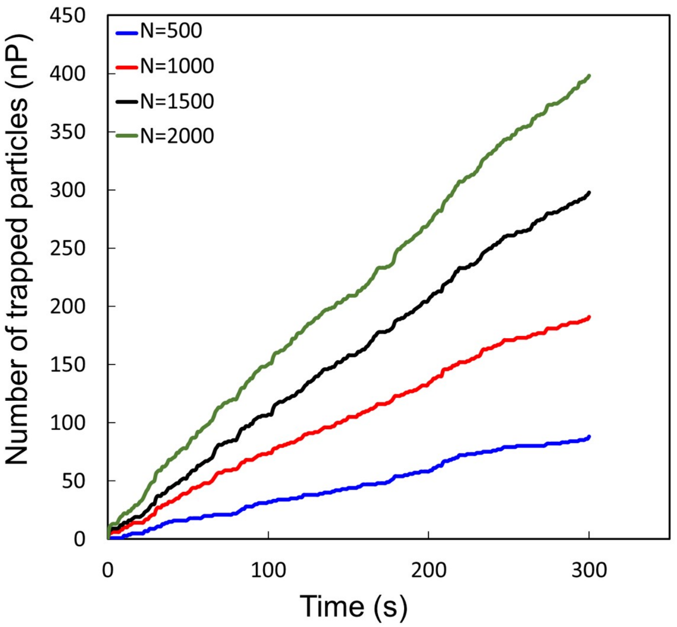

Figure S2.2: Numerical results showing the number of trapped particles as a function of time for different number of particles that are used in the simulation.

S3 Simulation details for the interdigitated electrodes

The following are the simulation results that are computed for the interdigitated electrodes. Figure S3.1 shows the tangential electric field distribution across the surface region of the electrodes, whose magnitude is highest at the platinum node and is sensitive to the increase in the concentration of hydrogen peroxide that fuels the electrocatalytic reaction. Consequently,

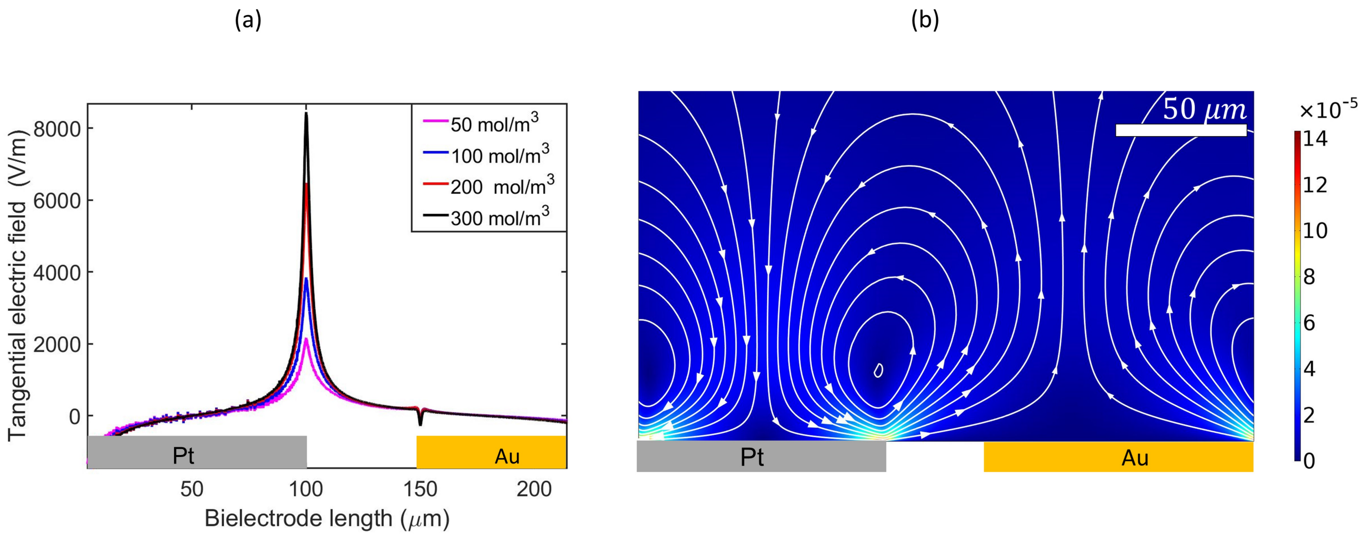

Figure S3.1: (a) Tangential electric field distribution across Pt-Au interdigitated electrodes (b) Velocity field streamlines for the platinum-gold interdigitated electrode driven by the induced electric field. The velocity magnitude is given in m/s.

the induced dielectrophoretic force is maximum at the surface of the platinum electrode Figure S3.2) and dominates over the drag and electrophoretic force at the surface region of the interdigitated electrodes (Figure S3.3).

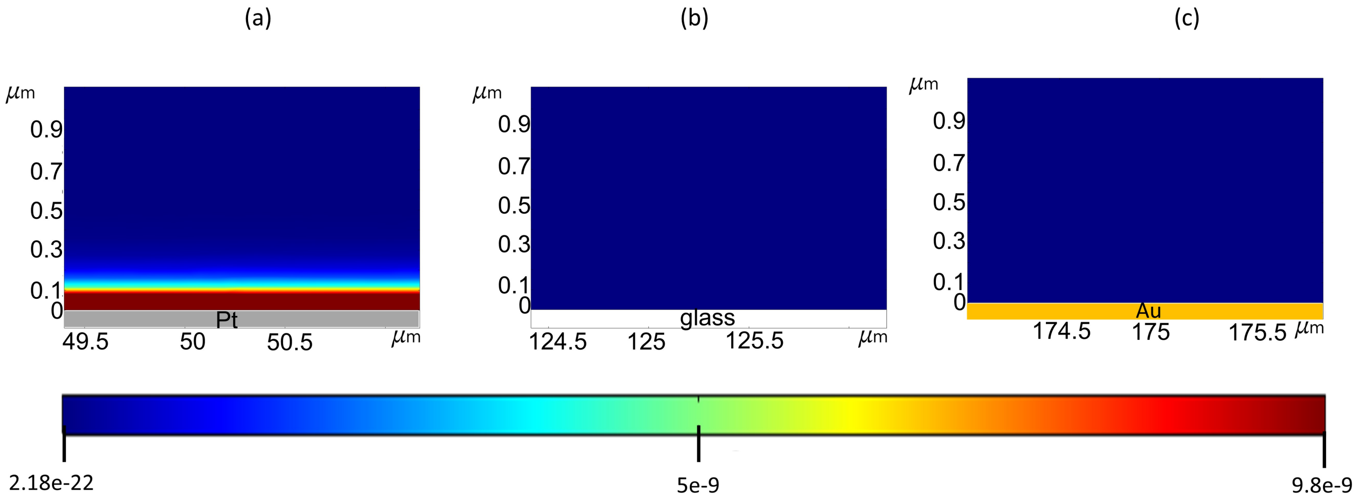

Figure S3.2: 2D surface plot for the dielectrophoretic force plotted around the center of (a) Platinum (b) Glass (c) Gold. The unit of the force is N.

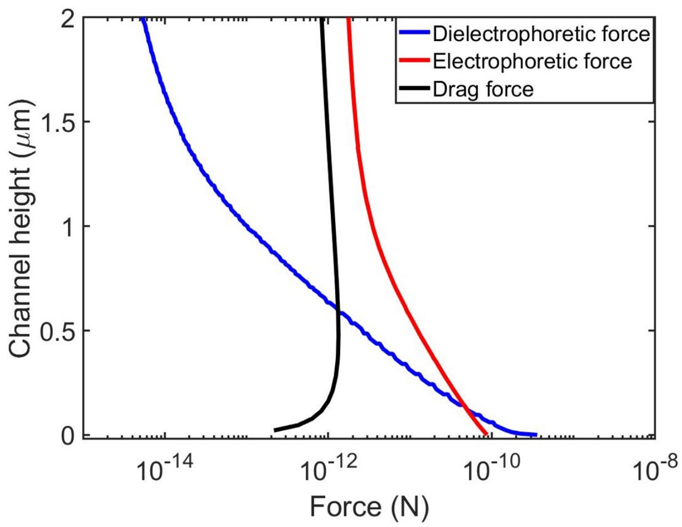

Figure S3.3: Variation of the forces acting on the particles along the channel height from the surface of the interdigitated electrode

S4 Brownian force

Brownian movements were observed for the particles in the absence of the drag, and electrophoretic forces during the off-mode where the trapped particles break apart and slowly

disperse into the bulk fluid medium. The Brownian force is given by[1]

$$\mathbf{F}_B = \xi\sqrt{\frac{12\pi k_B \eta T r}{\Delta t}} \tag{S4.1}$$

Where ξ is a dimensionless vector of independent, normally distributed random numbers, k_B is the Boltzmann's constant ($k_B = 1.38 \times 10^{-23}$ J/K), η the fluid viscosity, r the particle radius, Δt is the timestep size ($\Delta t = 1$ s), T is the absolute temperature of the fluid ($T = 293$ K).

The resultant Brownian force acting on the particles is determined to be approximately 8.85×10^{-15} N.

S5 Supplementary movies

5 supporting movies are provided that illustrate the particle trapping and accumulation dynamics for the bimetallic and interdigitated electrodes.

Movie 1 shows the transport of colloidal particles by the catalytically induced flow field from platinum to gold. Movie 2 shows the time lapse for particle trapping and accumulation at the junction of the Pt-Au bimetallic electrode. Movie 3 shows the accumulation of particles on the surface of the platinum electrode during the connected mode for the interdigitated electrodes. Movie 4 shows the accumulation of particles into clusters at longer time periods. Movie 5 shows the behavior of the particles under the disconnected mode of operation. The trapped particles break into singlets, and slowly disperse back into the bulk region.

References

(1) Kim, M.-m.; Zydney, A. L. Effect of electrostatic, hydrodynamic, and Brownian forces on particle trajectories and sieving in normal flow filtration. *Journal of Colloid and Interface Science* **2004**, *269*, 425–431.
